# Special Issue: Practical Applications of Metal Complexes

**DOI:** 10.3390/molecules20057951

**Published:** 2015-04-30

**Authors:** Iztok Turel

**Affiliations:** Faculty of Chemistry and Chemical Technology, University of Ljubljana, Večna pot 113, SI-1000 Ljubljana, Slovenia; E-Mail: Iztok.Turel@fkkt.uni-lj.si

**Keywords:** catalysts, photophysical properties, natural products as ligands, macrocycles as ligands, metal based drugs, metal drug interactions, radiopharmaceuticals, photodynamic therapy, magnetic properties, self-assembly

## Abstract

In 1913 Alfred Werner received the Nobel Prize in Chemistry for his work that was of great importance for the development of coordination chemistry. In the years that followed numerous complexes consisting of metal ions and organic ligands were isolated, thus building a strong connection between inorganic and organic chemistry. Coordination compounds have many interesting properties which find diverse applications in numerous aspects of human life. Fourteeen contributions were received for this Special Issue covering very different aspects of metal complexes and their practical applications. The highest number of manuscripts deals with the biological activity of complexes which might potentially be used in the clinical practice. Authors have tested their cytotoxicity, antibacterial activity and enzyme inhibition. Their optical properties were studied in view of their potential use in photodynamic therapy. Moreover, optical properties could also be used for bioanalysis. It is also known that metal complexes are useful catalysts and a few such examples are also described herein. Many other interesting properties and facts about the isolated and described complexes are also reported (radioactivity, design of metal-organic frameworks, *etc.*).

Metal complexes consist of a central metal atom or ion which is called the coordination centre and a surrounding array of bound molecules or ions, which are called ligands. It is clear that a huge number of very versatile metal-ligand combinations are possible. A brief analysis of the contributions revealed that ten d-block elements were reported in these studies (Fe, Ni, Cu, Zn, Tc, Ru, Rh, Ir, Pt and Au) but two studies describe also complexes of lanthanides and actinides. Among the main group elements only bismuth complexes were studied. Very versatile ligands were used in these contributions including monodentate (N-, P-, O-, S), bidentate (N,N-, O,O-, N,S-), and also multidentate examples. Organometallic compounds are also included in some of the studies. Metal complexes vary in numerous properties (colours, photophysical characteristics, magnetism, reactivity, biological activity, catalytic behaviour, structure, *etc.*), and many of these are described by the authors who contributed to this issue and are briefly described in this editorial.

In the late 1960s Rosenberg serendipitously discovered that the platinum complex cisplatin (*cis*-[PtCl_2_(NH_3_)_2_]) exerts anticancer activity [1]. Its clinical use was approved in 1978 and nowadays cisplatin is one of the world`s best-selling anticancer drugs [2,3] and surely the best-known metal- based drug. The great clinical success of cisplatin was a strong impetus to search for further novel platinum complexes that might also be used as drugs. Unfortunately, even cisplatin is not an ideal drug and has many disadvantages. Among others, it is not active against all kinds of cancer and many tumors develop resistance after the first treatment. It also exhibits numerous side effects (e.g., nausea, vomiting, loss of sensation in the extremities, bone marrow suppression, nephrotoxicity). For these reasons many groups have been trying to prepare compounds with better properties and a few other platinum complexes were introduced in the clinical practice. Two contributions dealing with platinum compounds can be found in this special issue. Although platinum-based drugs have been widely studied, exact knowledge of the mechanisms governing their accumulation in cells is still lacking. Casini *et al.* presented an overview of the information available on the cellular accumulation of platinum compounds (but also for some Ru, Au and Ir compounds) [4]. Piccialli *et al.* report the synthesis of four novel platinum-substituted adenosine complexes and also tested their cytotoxic behaviour [5].

The platinum success story has also triggered intensive research on other metal complexes (e.g., Ti, Ru, Ga) with potential anticancer and also other biological activities. At the moment none of the non-platinum complexes is in clinical use as an anticancer drug, but many of them are (or were) used in clinical trials. In the last decades extensive research has been performed on ruthenium complexes as two compounds, namely NAMI-A ([imH]trans-[RuCl_4_(dmso-S)(im)], im = imidazole) and KP1019 ([indH]trans-[RuCl_4_(ind)_2_], ind = indazole), have entered clinical trials [6,7]. A lot of work was also done on half-sandwich organoruthenium(II) complexes in which various ligands are coordinated to the central metal ion [8,9]. In the contribution of Hartinger *et al.* [10] organoruthenium complexes with thiourea were prepared and characterized. Compounds were also tested in urease inhibition tests where some of the compounds showed moderate activity.

It is well-known that metal ions and complexes interact with DNA [11]. It is also widely accepted that the interaction with DNA is crucial for the activity of cisplatin. Due to this fact several metal-based anticancer drugs were designed to interact primarily with DNA and were also mostly tested for such interactions. However, it later became clear that other targets are more important for many active metal-based drugs [12]. Still, the interactions with DNA may be used for other purposes, for example in bioanalysis. Metal complexes have thus been applied to probe both structural and functional aspects of nucleic acids [13]. Hui Wei *et al.* prepared a review on the recent progress of ruthenium polypyridine complexes as signal transduction elements and oligonucleotides as target recognition elements [14]. In this contribution electrochemistry, electrochemiluminescence and photoluminescence are mainly discussed.

Several promising families of metal complexes have been developed for the use in photodynamic therapy in the last few years [15]. Salassa *et al.* [16] present a paper on ruthenium photoactivatable complexes. They have prepared and characterized four novel organoruthenium complexes that can photorelease pyridyl ligands. Moreover, computational methods were used to obtain insights on the singlet and triplet excited states of the complexes.

Ferrocene was first prepared unintentionally in 1951. R. B. Woodward and G. Wilkinson deduced its sandwich-like structure based on its reactivity. Wilkinson later shared a Nobel Prize for his work on metallocenes and other aspects of organometallic chemistry. Several substituted ferrocenes have been isolated in last decades and the Jaouen group successfully attached the ferrocene fragment to tamoxifen, [17] an estrogen receptor modulator, which is a drug used for the treatment of breast tumours. The modification of tamoxifen by the introduction of a ferrocene fragment led to ferrocifen, which is also highly cytotoxic. In this Special issue, Jaouen *et al.* prepared ferrocenyl derivatives of diethylstilbestrol. Compounds were prepared through atypical McMurry cross-coupling reactions leading to a new series of potent antiproliferative compounds [18]. Similar strategies of coupling an organic drug with an organometallic entity that can potentially generate a more powerful drug was also used by Therrien *et al.* [19]. They have prepared six novel Rh(III) and Ir(III) metalla-rectangles prepared by a self-assembly strategy using the embelin-derived metalla-clips. Embelin is a phenolic lipid extracted from plants and it was shown that prepared metal complexes show an exceptional selectivity for cancerous over noncancerous cell lines. Molecular self-assembly is one of important concepts typical for supramolecular chemistry [20].

From the pioneering work of A. Werner a tremendous growth of coordination chemistry took place giving us an understanding of the synthesis, structure and reactivity of complexes and materials. In the last few decades novel branches of coordination chemistry have evolved, for example metal-organic frameworks (MOFs) and supramolecular coordination complexes (SCCs) [21]. Both share the design of metal nodes linked by ligands and can be defined as metal-organic materials (MOMs). MOFs are compounds in which metal ions are coordinated to often rigid organic molecules to form one-, two-, or three-dimensional structures. Such compounds can be porous and could be used for the storage and purification of gases, energy storage, purification, in catalysis, as sensors *etc.* Lanthanide metal-organic frameworks (Ln-MOFs) also present interesting photophysical properties. Wen-Wen Dong *et al.* have prepared and characterized three novel Ln-MOFs (with La(III), Ce(III) and Pr(III)) and studied their luminescent properties [22].

Quinolones are synthetic antibacterial agents broadly used in clinical practice. They easily form metal complexes with many metal ions [23]. The contribution of Jelikic-Stankov *et al.* deals with the speciation of Cu^2+^, Ni^2+^ and Zn^2+^ ions in human blood plasma in the presence of various fluoroquinolones under physiological conditions. The results of their computer simulation are important for better understanding of the role of metal complexes in the activity of these drugs [24]. The antibacterial activity of copper against *B. glumae*, which is a Gram-negative bacterium affecting humans but also plants (rice) was studied by Sun *et al.* [25]. They have found copper has a strong antibacterial effect against *B. glumae* (by causing DNA and membrane damage) and could therefore be used to reduce the risk of bacterial contamination and infection.

In the 1980s Marshall and Warren showed that the bacterium *Helicobacter pylori* (*H. pylori*) is the cause of most peptic ulcers [26]. This reversed the medical doctrine that ulcers are caused by stress, spicy foods and too much acid. Their findings were so important that they received Nobel Prize in Physiology or Medicine in 2005 for this work. It is very difficult to eradicate *H. pylori* and various approaches and combinations of drugs have been tested [27,28]. Contemporary treatments contain three to four drugs, frequently also a bismuth compound. Bismuth compounds have been used in medicine for several centuries. In the contribution of D. M. Keogan and D. M. Griffith the current and potential applications of bismuth-based drugs are extensively reviewed [29].

Radionuclides can be used in the processes of nuclear waste remediation, as catalysts, for radioactive labeling and more. Technetium complexes are for example extensively used as radiopharmaceuticals [30,31]. In the last 10 years, around 1,000 papers have been published on the preparation and characterization of technetium complexes or the use of ^99m^Tc as a radiotracer or radio-emitter in nuclear medicine. P. Elizondo Martinez *et al.* have prepared an extensive review on the coordination and organometallic chemistry of the actinides and Tc [32].

Without any doubt catalysis is one of the most important applications of metal complexes. Many Nobel prizes have been awarded for discoveries in catalysis, and in this century three Nobel prizes have already been awarded for catalytical processes in which metal complexes are involved. In 2001 W.S. Knowles, R. Noyori and K.B. Sharpless received the Nobel Prize for their development of catalytic asymmetric synthesis. In 2005 Y. Chauvin, R. H. Grubbs and R. R. Schrock received the Nobel prize for the development of the metathesis method in organic synthesis. Finally, in 2010 R. F. Heck, E. Negishi and A. Suzuki have also received the most prestigious scientific award for the development of palladium-catalyzed cross coupling [33]. All these discoveries were extremely important for chemical and pharmaceutical industry for preparation of products like drugs, plastics and many other products. In this Special Issue two contributions deal with catalysis. Vicic *et al.* [34] describe new paramagnetic nickel complexes. It was found that the complexes are active for the cross-coupling of alkyl electrophiles (especially with ethyl 2-bromobutyrate) with alkyl Grignard reagents. The second contribution by Clark *et al.* reports synthesis, immobilization and catalytic activity of a copper(II) complex with a chiral bis(oxazoline) [35]. The complex was immobilized onto the surface of a mesoporous carbonaceous material and the material acted as a selective and enantioselective heterogeneous catalyst in the kinetic resolution of hydrobenzoin.

Finally, I would like to thank the contributing authors who enthusiastically performed their work and prepared the manuscripts. Additionally, I would like to thank to the evaluators who reviewed the papers and contributed to their quality and last but not least, to the editorial staff of Molecules for their initiative for this Special Issue and for their support along the entire process. Now, I can only hope the readers will enjoy and learn something new from this interesting field.
